# Supplementary material for: Patterns of Intron Gain and Loss in Fungi
Source: PLoS Biol. 2004 Nov 30;2(12):e422. doi: 10.1371/journal.pbio.0020422 (PMC532390; doi:10.1371/journal.pbio.0020422)
Supplement: Table S1 — Also available at http://genes.mit.edu/NielsenEtAl/. (4.3 MB ZIP). [file pbio.0020422.st001.zip › NielsenEtAl/html/375.html]

AN7423.1.NCU03606.1.MG00685.1.FG10122.1


```
 CLUSTAL W (1.82) Multiple Sequence Alignments - Introns Inserted


Sequence 1: NCU03606.1	610 aa
Sequence 2: MG00685.1	610 aa
Sequence 3: FG10122.1	607 aa
Sequence 4: AN7423.1	606 aa
Alignment Length: 621 aa
Number Identitical Residues: 258 aa
Alignment Score (without introns) 14118


MG00685.1 	MADAAERQITRGAIA2AIFNDPEGVKTRFPVPVLQCLQVKLLGQQPN-AGAAERYRVVLS
NCU03606.1	MAG----QITQGALD2AMFNDPDRAQQQFPVPILQCLQIKTLDSKNGGAGATERFRIVLS
FG10122.1 	MSAA--EALSQGALD2VIFNDPDKATKLFPVPVLQCLQVKQMAPSAQ---GGDRFRLVMS
AN7423.1  	MASQ-ASHVSVGALS2VPSGIFDATKPQISEPIVQCVQVKPLPPQAN---NQERYRVVFS
          	*:      :: **:  .  .  : .   :. *::**:*:* :  .       :*:*:*:*

MG00685.1 	DVDNYIQCMLATQ~ANHVIHDDQLQRGCIVRVKSYQANTVKGKS2VLVLLDLEVIQSLGV
NCU03606.1	DLKNYVQCMMATQ~TNHLVHDGLLQRGCIVRLKQYQAQCLKGKN2ILIVLDLEVIQSLGC
FG10122.1 	DGQHYVQTMLATQ~ANHVVHDNKLVRGCFARIKQYTPNNLKGKN2ILVILDIEVIESLGI
AN7423.1  	DISNYVQTMLAIQ1ANHFVTDGLLRKGCFVRLKQFQANSVKGKK2ILIVLDLEVLKELGE
          	* .:*:* *:* * :**.: *. * :**:.*:*.: .: :***. :*::**:**::.** 

MG00685.1 	HEKIGEPASIEAK-AENTTN--TTIGGSGFYGTKTEP--DVKPQIRSQMPTRNTGGGGAS
NCU03606.1	PEKMGDPQPLGPRSAEPQQN--PNLGSTGFYGVKSEPTQDTKPQFPRQMPSRNASGG---
FG10122.1 	QEKIGEPSAFDAKPAGQE----SAIAGGDFYGVKKEESKTQPQQFQQQQQSMPSRPA---
AN7423.1  	AEKLGEPKPLESKAEEEEKPQPTTISSNGFYGAKAQN---APSQISSRAQHTRPMQG---
          	 **:*:* .: .:.      ... :.. .***.* :       *:  :     .  .   

MG00685.1 	HGPGNVNIYPIESISPYQHKWTIKARVSQKSDIRTWHKASGEGKLFSVNLLDETGEIKAT
NCU03606.1	--QGSSTIYPIEGLSPFSHKWTIKARVTSKSDIKTWHKASGEGKLFSVNFLDESGEIRAT
FG10122.1 	-MHAGSNIYPIEGLSPFAHKWTIKARVTAKSDIKTWHKATGEGKLFSVNLLDESGEIKAT
AN7423.1  	--SAPATIYPIEAISPYAHKWTIKARCTSKSPIKTFHGRSGDGTLFSVNLLDDSGEIRAT
          	   .  .*****.:**: ******** : ** *:*:*  :*:*.*****:**::***:**

MG00685.1 	GFNDQCDKFYDILQEGQVYYISTPCRVQMAKKQFTNLPNDYELTFEDGTQIEK~AEDQSS
NCU03606.1	GFNDQVDQFYDLLQEGQVYYISTPCRVQLAKKQWSNLPNDYELTFERDTVIEK~AEDQTS
FG10122.1 	GFNDQCDALYDQLQEGSVYYISTPCRVQLAKKQFSNLPNDYELTFERDTVVEK~AEDQTN
AN7423.1  	GFNEQCSALYDLFQEGEVYYISSPCRVQIAKKQFTNLNNDYELTFERDTLVEK0AEEQND
          	***:* . :** :***.*****:*****:****::** ******** .* :** **:*..

MG00685.1 	VPQVRFNFCNIQELQEVEKDATVDVIGVLKDVADVTQITSKASGKFFDKRELTLVDDSGY
NCU03606.1	VPQVRFNFVNIQELQDVERDATVDIIGVLKEVQEVTQIVSKTTQKPYDKRELTLVDNTGY
FG10122.1 	VPQVRFNFCSIQELQSVEKDNTVDVIGVLKEVGEIGDITSKKDGRPFQKRELTLVDDTGF
AN7423.1  	VPQVRFNFTTIGDLQSVEKDTTIDVIGVLKDVGETTQIVSKTTKKPYDKRELTLVDNTGF
          	******** .* :**.**:* *:*:*****:* :  :*.**   : ::********::*:

MG00685.1 	SVRMTIWGKTAQNFDAKSESVVAFKGAKVGDFGGRSLSLLSSGTMTVDPDIPEAHRLKGW
NCU03606.1	SVRCTIWGKTATNFDAQPESIVAFKGTKVSDFGGRSLSLLSSGTMAIDPDIPEAHHLKGW
FG10122.1 	SVRVTIWGKNANSFDAAPESVVAFKGTKVSDFGGKSLSLLSSGTMTVDPDIPDAHRLKGW
AN7423.1  	SVRLTIWGTTAMNFAASPESVVAFKGVKVSDFGGKSLSLLSSGSVTVDPDIEEAHRLKGW
          	*** ****..* .* * .**:*****.**.****:********::::**** :**:****

MG00685.1 	FDSSGRNDNFSTFN-SNSVAGATGRQDQILTIHKVKEDNLGVDDV-VYFALKATVVYIRQ
NCU03606.1	YDSSGRNNTFATHNNMQTLGGATGRKDDAKTISQVKEENLGTNEAPDYFALKATVVFIKQ
FG10122.1 	YDSAGRTDTFATHQNMASVGSATGRKEDLKTIAQVKDENLGMDDQ-AYYTIKATIVFVKQ
AN7423.1  	YDAQGRNENFASHASSVGTMSATKR-DQLKTIAQVREEQLGMSEEPSYFTLRATVVYIKQ
          	:*: **.:.*::. .     .** * ::  ** :*::::** .: . *::::**:*:::*

MG00685.1 	ENFAYPSCLNEGCSKKVTDLGDGSWRCEKCDVNHPRPEYRYIM2SVNVNDHTGQLWLSCF
NCU03606.1	DNFAYPGCRSEGCNRKVTDMGDGTWRCEKCQINHDRPQYRYIM~SVNVNDHTGQLWLSCF
FG10122.1 	ENFCYAACLSQGCNKKVTQMPDGTWQCEKCNLSHEKPEYRYVL~SLNVADHTSHQWLTGF
AN7423.1  	DNLCYPACLSEGCNKKVTEVDPGQWRCERCDKTHPRAEYRYIM~LVNVSDHTGQLYLNCF
          	:*:.*..* .:**.:***::  * *:**:*: .* :.:***::  :** ***.: :*. *

MG00685.1 	DDVGRIIMGKSADELMALKDENFEAFTREFENANCRKLSFRCRAKMDTFGDNQR2VRYQV
NCU03606.1	DDTARVIMGKSADELMEIRETDETRLPAEFEQANCRKLNFRCRAKMDTFGEQQR2IRYQV
FG10122.1 	DDFGRQVMGRTADEMMELKENDDTKLTAAFEEANCKKFTFRCRAKMDNFGEAQR2IRYQV
AN7423.1  	DDVGRLLLGMSANQLMELPQNDGKALGNVIQNATCRTWTFGCRAKIDHYGDQQR2IRYQV
          	** .* ::* :*:::* : : :   :   :::*.*:. .* ****:* :*: ** :****

MG00685.1 	MGATKMDWKSEAARLADLIKQFNI---
NCU03606.1	MSVAPLDYKMEGNKLNELINSYNQMSM
FG10122.1 	MSVTPLDFKSEGTKLAELIKQYENTSL
AN7423.1  	SYAKPVNYSEEASRLADIIDSYSIS--
          	  .  :::. *. :* ::*..:.
```
